# Prevalence of Obesity in School-Going Children of Karachi

**DOI:** 10.1371/journal.pone.0004816

**Published:** 2009-03-24

**Authors:** Haider Javed Warraich, Faisal Javed, Mohammed Faraz-ul-Haq, Fariha Batool Khawaja, Sarah Saleem

**Affiliations:** 1 Medical College, Aga Khan University, Karachi, Pakistan; 2 Department of Community Health Sciences, Aga Khan University, Karachi, Pakistan; London School of Hygiene and Tropical Medicine, Peru

## Abstract

**Background:**

Obesity is an emerging problem in Pakistan. The authors sought to determine prevalence of obesity and malnutrition in school-going children, from grades 6^th^ to 8^th^ of different schools of Karachi and assess associations that affect the weight of the children.

**Methodology/Principal Findings:**

A cross sectional study design with children studying in grades 6^th^ to 8^th^ grade, in different schools of Karachi. We visited 10 schools of which 4 consented; two subsidized government schools and two private schools. A questionnaire was developed in consultation with a qualified nutritionist. Height and weight were measured on calibrated scales. A modified BMI criterion for Asian populations was used. Data was collected from 284 students. Of our sample, 52% were found to be underweight whereas 34% of all the children were normal. Of the population, 6% was obese and 8% overweight. Of all obese children, 70% belonged to the higher socio-economic status (SES) group, while of the underweight children, 63.3% were in the lower SES. Amongst obese children in our study, 65% ate meat every day, compared to 33% of normal kids.

**Conclusion:**

Obesity and undernutrition co-exist in Pakistani school-children. Our study shows that socio-economic factors are important since obesity and overweight increase with SES. Higher SES groups should be targeted for overweight while underweight is a problem of lower SES. Meat intake and lack of physical activity are some of the other factors that have been highlighted in our study.

## Introduction

Overweight and obesity are a global pandemic. According to a WHO report, there are 1 billion overweight people in the world, of whom 300 million are obese[1]. Findings of the National Health Survey, Pakistan, 1990–1994, found that the prevalence of obesity for adults aged 25–64 from low, middle to high socioeconomic status (SES) was 9%, 15% and 27% for rural areas and 21%, 27% and 42% for urban areas respectively[2]. Prevalence of undernutrition, stunting and wasting has been studied widely in Asia; 70% of the world's malnourished children live in Asia[3]. A study done in rural Pakistan found 26% of the sample population children were wasted, 55% were stunted and 15% were both wasted and stunted[4].

Pakistan is a country in transition and now faces double burden of coexistent overnutrition and undernutrition. Obesity is becoming an increasingly prevalent problem in Pakistan, as it has in other developing countries, with undernutrition remaining a problem simultaneously. Such has been observed in comparable developing countries such as Egypt [5] and India [6].

New recommendations for the Asia-Pacific population suggest that a Body Mass Index (BMI) of greater than 23 for overweight and greater than 25 for obesity be used, as opposed to the usual limits of greater than 25 and greater than 30 used internationally for overweight and obesity respectively[7]. A recent study demonstrates that a quarter of the population of Pakistan would be classified as overweight or obese with the use of Indo-Asian-specific BMI cutoff values[8]. This is significant because the risk of being overweight in adulthood has a positive correlate with being overweight in childhood and adolescence[9]. The prevalence of obesity in adolescents in Pakistan is already significant, with one study putting the prevalence of adolescents with a BMI greater than 25 at 18%[10], which is comparable with figures in the West. However, due to our different social and cultural setting, the associations leading towards obesity studied in the West cannot be applied on our population as for example, socioeconomic conditions are directly proportional to overweight as opposed to the Western World[11].

Childhood obesity and overweight is an emerging problem in Pakistan[12]. As a developing nation, it is important to address this issue to decrease the disease burden [13]. Teaching better practices to children can be one way to take care of this concern[14]. The authors are of the opinion that obesity is no longer confined to the West and is a problem that needs to be emphasized upon in developing countries as well[15].

Extensive research has been done into obesity in neighboring India. Just like Pakistan, the double burden is increasing; a study done on school children in urban Madras found the number of overweight boys to be 17.8%, and girls 15.8%. In affluent cities of India, prevalence of obesity reach the levels of industrialized countries, with values increasing with socioeconomic class [16], [17]. This should be seen in the context of the double burden –a WHO study found 30–70% of Indian adults to be underweight, greater than the proportion in Sub-Saharan Africa [18].

Thus targeting children with interventions is the best and most cost effective way of dealing with obesity and the diseases it is responsible for. Obesity is not only associated with cardiovascular disease but studies have suggested that it plays a role in the initiation of disease early in life[19]. A study which followed up school children with essential hypertension found that those children that reported high blood pressure had lower levels after a 6 and 9 month follow up[20]. This might show an increase in positive health attitude of children after being sensitized to their hypertensive status emphasizing the importance of such studies in spreading awareness about healthy attitudes. School-based interventions have been encouraged by the American Diabetic Association to control pediatric overweight by promoting physical activity and nutrition education[21]. Our project focused on assessing the nutritional status of school going children in grades 6^th^ to 8^th^ in different schools of Karachi. We did so following current BMI guidelines for the Asia-Pacific region. To assess their dietary habits, brief interviews were conducted with the children.

## Methods

### Study Design

A cross sectional study design with children studying in grades 6^th^ to 8^th^ grade in four schools of Karachi.

### Setting

We visited 10 schools on a spectrum determined by their fee structures. Of the 10, 6 schools refused consent because of ongoing school examinations. Of the four schools that consented, two were subsidized government schools and two were private schools.

### Participants

Consenting male and female students of grades 6^th^ to 8^th^ were included in the study. We asked ten schools for permission allowing us to conduct our study on their students. From the thousands of schools in Karachi, the authors selected ten well-recognised schools, whose monthly fee ranged from Rupees 5,000 to subsidized schools that offered free education. There was no mechanism for randomization in place. Schools were offered the incentive of lectures administered by medical students to increase educate children about appropriate nutrition and the need for physical activity. Four out of the ten consented; six schools refused, citing ongoing exams as the reason. Consent forms, in both English and Urdu, for all students of grades 6^th^ to 8^th^ were signed by either one of the parents. Two days after that we went to collect consent. Data was collected from students who had gotten permission.

### Data Sources

#### Measurement

**Figure 1:**
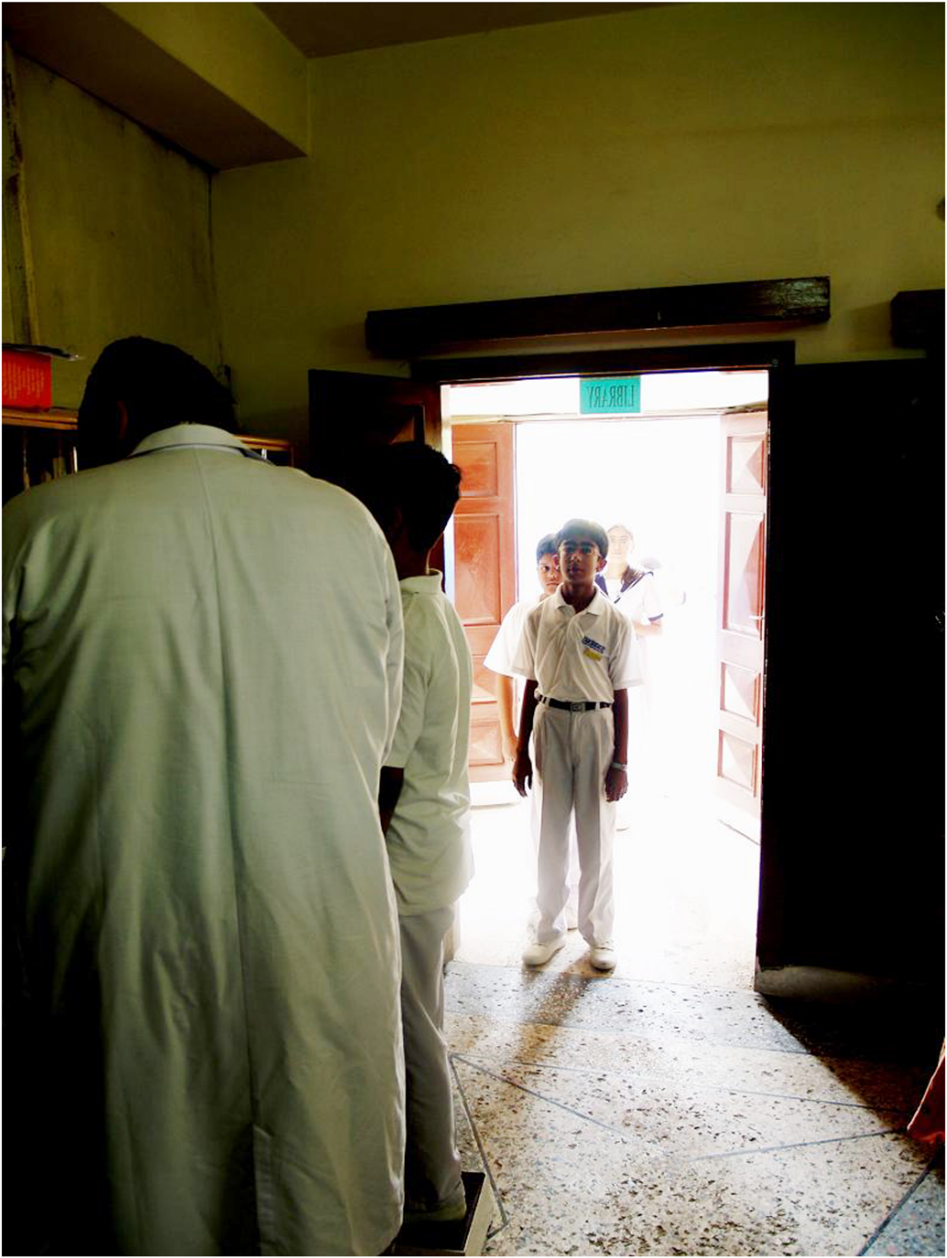
Faisal Javed measures the height and weight of a child at a private school in Karachi. Photo Courtesy: Haider J Warraich.

Height and weight of each student were measured using calibrated scales. Body Mass Index (BMI = weight (kg)/height^2^ (m^2^)) was calculated for each student. Precautions were taken to make sure all recordings were accurate and precise. All measurements were conducted by one student (figure 1).

#### Interviews

**Figure 2:**
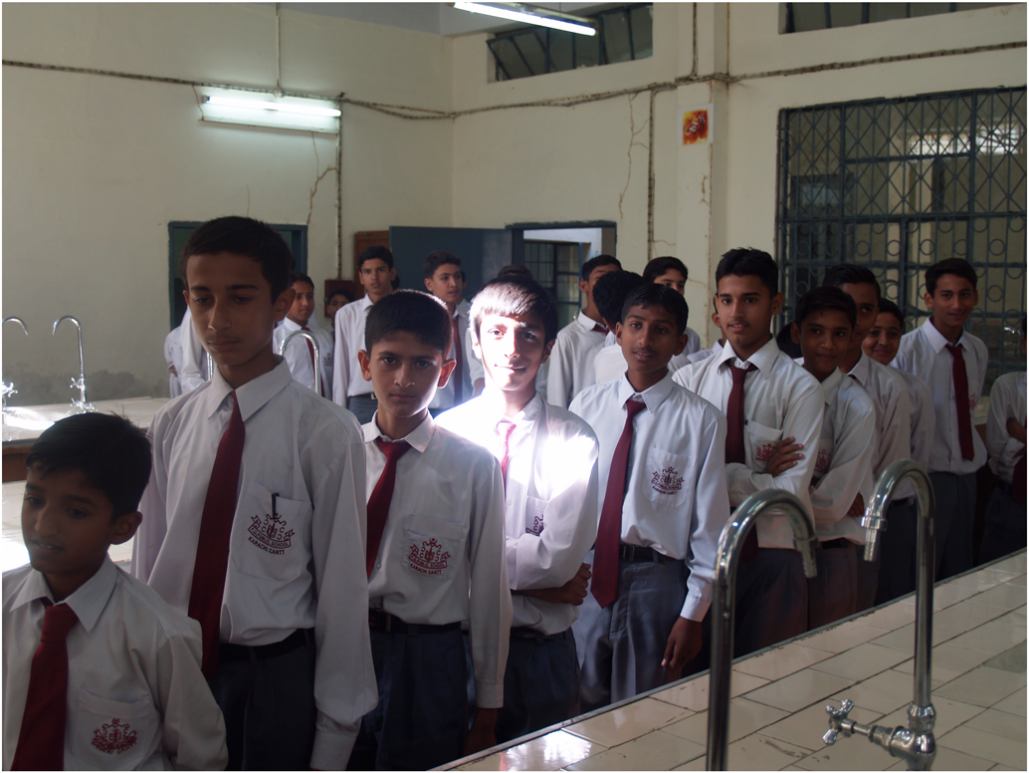
Children line up for measurements and interviews at a government-run public school in Karachi. Photo Courtesy: Haider Warraich.

Five-minute long interviews were conducted by the medical students involved in the study. Interviews took place during the recess so that children's time was not wasted. It was optional for the child whether he or she wanted his or her name on the form. The questionnaire was pretested on children visiting the Community Health Centre, Aga Khan University. The interviewers were all medical students and the interview was conducted in the local language, Urdu (figure 2).

#### Questionnaire

A pilot questionnaire was developed in consultation with a qualified nutritionist at the Aga Khan University. Questionnaire consisted of **four** sections. **Section 1** assessed demographic information: name, age, gender, school name, grade, height (meters) and weight (kilograms). **Section 2** measured socioeconomic status on the basis of area of residence, school fee structure, type of house, ratio of number of rooms to number of people living in the house, the type of transportation they have and their number, possession and number of household appliances including air conditioner, computer, microwave oven and television. The variables were based on previous studies in Pakistan [18]. **Section 3** evaluated dietary intake including questions on number, quantity and type of regular meals taken. With regards to intake of specific foods, it was inquired whether meat[15], fruits, milk, rice and junk foods[22] including cold drinks, burgers, pizza, chocolates, chips, ‘samosay’ and ‘pakoray’ were taken. A question also assessed parental awareness of child's nutritional status[22]. **Section 4** assessed the level of physical activity including level of physical activity[23], including questions on number of hours of watching TV/playing video games per day[24], number of times a sport is played per week (defined as >30 minutes of activity) and punctuality in school physical education classes[25].

#### Study Size

Keeping confidence level at 95%, anticipated population proportion of overweight at 15% and absolute precision error at +/−5%, our estimated sample size was 196. Gender differences were not considered in our calculation. However, we collected data from all consenting students from within the selected grades and schools.

#### Quantitative Analysis

BMI of children was calculated using height (meters) and weight (kilograms) of children. Socio-economic variables were assigned scores and based on the scoring system we formulated 2 groups i.e. high and low socio-economic status. Data was obtained to find correlate between SES and BMI, and high consumption of specific food items with BMI. Specific food items were given scores to divide into categories of high and low consumption: everyday intake was given 2 points, 1 point for intake after a gap of at least 2 days and 0 points for no intake at all. We performed chi-squared test of high vs. low consumption of junk food against different BMI categories.

#### Data Analyses

The data was entered and analyzed on the EPI-Data and SPSS version 10, statistical analysis programs. Chi-square test was used to determine the significance of association between the variables. Odds ratios were also computed to determine the strength of the association. There was no missing data.

#### Ethical Statement

The study was granted approval by the Ethics Review Committee of the Aga Khan University, Karachi. Consent forms, in both English and Urdu, for all students of grades 6^th^ to 8^th^ were signed by either of the parents of the children. Data was collected from students who had gotten permission.

## Results

Of 306 students given consent forms, 284 consented and participated in the study. Thus the response rate was 92.8%. There were more males than female participants (Table 1). We selected our population according to contiguous grades as opposed to age, which varied between 11 and 17 years of age; 92% of our population was between 11 and 14 years of age. There was no relationship between the BMI categories and age in our study.

**Table 1:** No. of children interviewed (height and weight also recorded) by Class and Gender.

Sex	Class			Total
	6th	7th	8th	
Male	67	54	54	175
Female	37	35	37	109
Total	104	89	91	284

Table 2 shows that the proportion of obese/overweight children increases with SES with the reverse being true for malnutrition. Of obese children, 71% were in the higher SES while 63.3% of underweight children were in the lower SES. For the relationship of SES against normal children and obese children, chi-square test was performed (Table 3).

**Table 2:** Frequency of BMI categories against socio-economic status.

SES	BMI Category				Total
	Underweight	Normal	Overweight	Obese	
Low SES	93	52	10	5	160 (56%)
High SES	54	45	13	12	124 (44%)
Total	147 (51.8%)	97 (34.1%)	23 (8.0%)	17 (6.0%)	284 (100%)

**Table 3:** Relationship of high socio-economic status with obesity (P = 0.066).

Socio-Economic Status	Normal vs. Obese		Total
	Obese	Normal	
Low	5	52	57
High	12	45	57
Total	17	97	114

According to our scoring system, 80% of all kids have a high consumption of junk food. Intake was regardless of SES although it had no relationship with nutritional status. For food variables, we performed chi-squared test on key food items against BMI categories. Of obese children, 65% ate meat every day, compared to 33% of normal kids. Meat consumption was high across the board; however, it was higher in children from higher socioeconomic class.

About 30% of all groups play cricket every day, except for obese kids, of whom only 6% did so. Of obese kids, 47% claimed to not play cricket at all. When we asked the children about their parents' opinion with regards to their eating habits, more than half of kids from all categories replied that their parents thought they were eating normally.

## Discussion

Of our population, 70% of all obese children belonged to the higher SEC, while of the underweight children, 63.3% were in the lower SEC. This finding is consistent with the view of previous studies that obesity in developing countries increases with socioeconomic class[26]. Of our total population, 51.8% of children were underweight, which is also consistent with previous studies done in urban areas. Only 34% of the entire population lay within the normal range (Table 2).

Meat consumption was high across the board; however, it was significantly higher in children from higher SES. Within overweight and obese kids, there was not a single report of children not eating meat at all. This was however, not true of many underweight children belonging to lower SEC, who reported not having meat at all. Meat intake has been found to be associated with obesity and its cardiovascular complications. Children in Switzerland showed a direct association between intake of meat products and overweight[27]. In Rome, obesity was found to be significantly associated with hypertension, and hypertensive school children had a higher intake of meat and eggs in their diet[28]. On top of this, knowledge about dietary risk factors for cardiovascular disease seems to be lacking. In Poland, few school children recognized salted meats as risk factors, though knowledge about other factors like smoking and alcohol misuse was satisfactory[29].

Lack of physical activity was found to be associated with obesity in children. Cricket was found to be the most popular sport to the exclusion of all the others, which were football (soccer), basketball, games involving running (like tag) and cycling. About 30% of all kids play cricket 7 days a week, except for obese kids, of whom only 6% do so. In fact, 47% of obese claimed to not play cricket at all.

The Online Medical Dictionary by Farlex defines ‘junk food’ as ‘any of various prepackaged snack foods that are high in calories but low in nutritional value’. According to our scoring system, 80% of kids have a high consumption of junk food. This bodes unwell for the future, as junk food may overtake conventional meals in causing obesity. Intake was seen to be regardless of SEC, which means that all children are at risk.

When we asked the children what their parents' opinion with regards to their eating habits was, more than half of parents of kids from all categories replied that their parents thought they were eating normally. This is contrary to the actual nutritional status of the child which could have been underweight, overweight or obese. This displays a need to educate parents, as well as children, about the basics of nutrition, a balanced diet and the ideal height and weight of the child.

Lack of randomization in school selection hampered the generalisability of the study. However, the schools that consented reflected a large variation in socio-economic status of children studying, which is reflective of Pakistani society. The study design also did not approach children with no access to schooling.

Obesity and undernutrition co-exist in Pakistani school-children. Our study shows that socio-economic factors are important since obesity and overweight increase with SES. Meat intake and lack of physical activity are some of the other factors that have been highlighted in our study. Obesity is an emerging problem in Pakistan and it should be studied in greater detail with larger, randomized, well-funded generalisable studies. With both underweight and overweight co-existing, any public health intervention should incorporate measures to alleviate both. Using schools to teach children better dietary practices and the benefits of physical activity would go a long way in helping developing countries such as Pakistan overcome the double burden of disease. Integrated approaches that promote intake of a balanced diet and increased physical activity to both underweight and overweight children would be more cost-effective than separate strategies for underweight and overweight children.
